# Comparative Quality Assessment of Five Bread Wheat and Five Barley Cultivars Grown in Romania

**DOI:** 10.3390/ijerph191711114

**Published:** 2022-09-05

**Authors:** Elena Moroșan, Ana Andreea Secareanu, Adina Magdalena Musuc, Magdalena Mititelu, Ana Corina Ioniță, Emma Adriana Ozon, Ionuț Daniel Raducan, Andreea Ioana Rusu, Adriana Maria Dărăban, Oana Karampelas

**Affiliations:** 1 Department of Clinical Laboratory and Food Safety, Faculty of Pharmacy, “Carol Davila” University of Medicine and Pharmacy, 6 Traian Vuia Street, 020945 Bucharest, Romania; 2 Department of Pharmaceutical Technology and Biopharmacy, Faculty of Pharmacy, “Carol Davila” University of Medicine and Pharmacy, 6 Traian Vuia Street, 020945 Bucharest, Romania; 3 “Ilie Murgulescu” Institute of Physical Chemistry, 202 Spl. Independentei, 060021 Bucharest, Romania; 4 Faculty of Pharmacy, “Vasile Goldiș” Western University of Arad, 86 Liviu Rebreanu Street, 310045 Arad, Romania

**Keywords:** *Triticum aestivum* L., *Hordeum vulgare* L., conventional plant breeding, nutritional knowledge, human health

## Abstract

Cereals whole grains contain vitamins, phytochemicals, antioxidants, resistant starch, and minerals with potential benefits to human health. The consumption of whole grains is correlated with a lowered risk of the most important chronic diseases, including type II diabetes, cardiovascular diseases, and some cancers. This study aimed to characterize and evaluate the content of five cultivars of wheat (*Triticum aestivum* L.) and five cultivars of barley (*Hordeum vulgare* L.) obtained by conventional plant breeding using crossing and selection methods. The novelty and the purpose of this research was to quantitatively and qualitatively analyze these ten cultivars from Romania and to show the importance of, and the changes produced by, crossing and selection methods when these are aimed at the physiological or morphological development of the cultivars. Studies based on gluten dosing; spectrophotometry using Bradford, fructan and protein dosing; Kjeldahl protein dosing; GC-MS/MS-protein and amino acid dosing; and identification of protein fractions using polyacrylamide gel electrophoretic method were conducted. This study demonstrates the possibility of developing future cultivars using conventional methods of improvement to modify the content and composition of nutrients to increase their health benefits.

## 1. Introduction

Consumption of wheat-based products is associated with a reduced risk of developing chronic diseases [1,2]. Recent studies regarding the primary potential health benefits of wheat-based products show that an increased consumption of wheat fiber and cereal whole grains improves intestinal transit, reduces obesity risks and cardiovascular diseases, prevents type-II diabetes and decreases the chances of developing some forms of cancer [3,4,5,6]. Carbohydrate fermentation in the colon promotes the development of the intestinal flora and improves the production of beneficial compounds such as short-chain fatty acids [7]. Additionally, wheat seeds contain polyphenols, carotenoids, ascorbic acid, vitamin E and phytosterols. These bioactive chemicals provide various biological activities, such as antioxidant, antidiabetic, antiinflammatory and antithrombotic properties [8,9]. Barley (*Hordeum vulgare* L.) is one of the oldest crops cultivated in the world with a natural tolerance and a highly adapted capacity [10]. Its use over time as a food source has also carried it through the malt, beer, and distillery manufacturing industries [11]. Barley seeds contain little fat, carbohydrates (mainly starch), and enough protein to provide the body with the necessary amino acids, minerals, vitamin E, polyphenolic compounds and soluble or insoluble fiber [12]. The endosperm is rich in B-glucans and plays an important role in the regulation of serum cholesterol and blood sugar, supporting the proper function of the cardiovascular system and controlling diabetes [13]. The high fiber content gives the effect of satiety, having a positive impact on weight control, but also on the transit of food in the colon [11]. In comparison with barley, wheat (*Triticum aestivum* L.) is one of the most widespread and important cereal crops, being a major source of feed for livestock and industry, and for nutrition [12].

In the last decade, great efforts have been made to find strategies that improve human public health. These have focused mainly on nutritional knowledge and information regarding dietary components [14,15,16,17,18,19]. In 2015, the most cultivated plants obtained by conventional plant breeding were soybeans (83% of crops), cotton (75% of crops), corn (29% of crops) and canola, a hybrid of rapeseed (24% of crops) [20].

In Romania, the modification of plant crops by crossing and selection methods is an important process in which higher hybrid varieties can be obtained. For this study, five cultivars of wheat (*Triticum aestivum* L.) and five cultivars of barley (*Hordeum vulgare* L.) obtained by crossing and selection methods of improvement, were purchased from the National Agricultural Research and Development Institute Fundulea. The selection of these cultivars was preferred due to their high consumption and their large cultivated areas—with Romania occupying sixth place at for these at the European level. Simultaneously, quantitative changes in the content of some nutrients in cereals can be correlated with certain diets in order to correct deficiencies or adjuvants for treating certain pathologies.

Improving the nutritional quality of wheat varieties plays an important role in the bakery industry. The ability to make quality dough depends on both the gluten content (the majority protein component of the wheat grain) and also on the content of fermentable carbohydrates (small molecule carbohydrates). The nutritional value of wheat flour is also provided by the content of essential amino acids, minerals, vitamins and fibers. Increasing fiber content is important for the diets of diabetic, obese, or constipated people. Increasing the gluten content improves the baking properties [21,22], but restricts the consumption by people with gluten allergies or intolerance [23].

Enhancing the nutritional qualities of barley varieties plays an important role in the beer industry because barley is the raw material for obtaining malt. In the technological process of obtaining beer, the quality of the raw materials is essential for obtaining a nutritious final product with special organoleptic characteristics [24,25].

Both types of cereal cultivars that are the subject of this study (wheat and barley cultivars) represent important raw materials for obtaining food products with a large market worldwide. Considering the current interest in finding ways to increase the nutritional value of food products, this work characterizes, from a qualitative viewpoint, some improved cereal cultivars that are provided from raw materials that are themselves rich in nutrients and that have special technological qualities for the food and beer industries. The novelty of the study consists in the characterization of new, improved cereal cultivars, especially in terms of the important nutrients designed for the quality of the final products derived from them—the bakery products obtained from the processing of wheat flour and the beer varieties obtained from malt. Both types of cereal cultivars represent important raw materials for food products with increased consumption among the population: bakery products and alcoholic beverages such as beer. These findings will help in filling the knowledge gap for the wheat and barley cultivars produced by crossing and selection methods.

## 2. Materials and Methods

### 2.1. Materials

Five wheat cultivars (*Triticum aestivum* L.) and five barley cultivars (*Hordeum vulgare* L.) obtained by crossing and selection methods were used in this study. The cereal cultivars were purchased from the National Agricultural Research and Development Institute Fundulea (Calarasi County, Romania). The Lugol’s reagent and bovine serum albumin (BSA lyophilized powder fraction V ≥ 98%) were purchased from Sigma Aldrich (Steinheim, Germany). The chemicals were used as received.

The characteristics of the analyzed cereals obtained from the National Agricultural Research and Development Institute [26] are represented in Table 1.

### 2.2. The Milling Process

The cereal seeds were ground using FOSS Cyclotec 1093 Mill laboratory equipment to achieve a fineness and uniform size of the particles. A Mettler Toledo XS205 DualRange analytical balance was used for the weighing of the sample.

### 2.3. Gluten Detection Method from Wheat

We moistened 10 g flour with 5 mL of purified water, it was kneaded to a non-sticky consistency and was then left to stand for 20 min. Then, it was washed for 30 min with purified water to remove the starch. The elimination of starch was complete when the residual water did not change the color of a drop of Lugol’s reagent. The gluten wet mass was weighed. The sample was then crushed and placed in a heat-resistant vessel in the oven at 105 °C and 760 mmHg, for two hours. After that, the dry mass of gluten was weighed.

### 2.4. Wheat Protein Content Using the Bradford Assay

The Bradford assay [27] is based on an absorbance shift of the dye Coomassie Brilliant Blue (CBB) G-250, by the binding of protein molecules. The absorbance of the dye–protein complex and the efficiency depend on the amino acid composition of the protein, and it has a maximum absorption at a wavelength of 595 nm.

An amount of 0.010 g Coomassie Brilliant Blue G was dissolved in 5 mL ethanol and 10 mL phosphoric acid. Then, it was brought to a final volume of 100 mL with distilled water. Before use, it was refrigerated and filtered. Tris-glycine buffer at pH = 8.3 was obtained as follows: 0.600 g tris (hydroxymethyl) aminomethane and 2.88 g glycine is dissolved in purified water and then diluted to 100 mL with the same solvent. Before use, it was made a 1:10 dilution with distilled water as follows: 20 mL tris-glycine buffer prepared above is brought to a final volume of 200 mL with distilled water. Bovine serum albumin (BSA) stock solution (1 mg/mL) was prepared as follows: 0.025 g BSA was put into a 25 mL volumetric flask. An amount of 10 mL tris-glycine buffer diluted at pH = 8.3 (or any other solvent used to dissolve the unknown sample) was added. The solution was stirred until the substance was solubilized and it was brought to a level of 25 mL with Tris-glycine at pH = 8.3 buffer. The test solution was prepared by weighing 50 mg wheat and 50 g barley. The samples were prepared in duplicate. An amount of 3 mL tris-glycine buffer was added over the first row of samples, and 3 mL distilled water over the second row. For efficient dispersion, the samples are placed on the sonicate bath for 5 min at 4 °C. Then, the samples were centrifuged for 30 min on ice, at 4000 rpm. The samples were kept in a refrigerator. The calibration curves were obtained using the dilutions shown in Table 2, which are made in ten Eppendorf vials of 1 mL volume.

**Figure 1:**
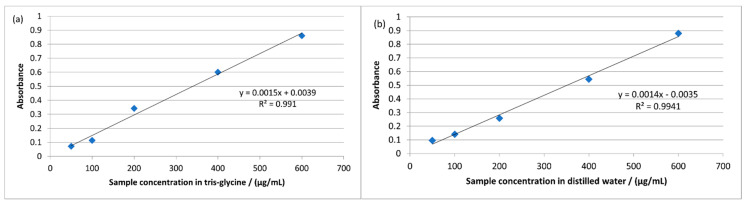
The calibration curves in (**a**) tris-glycine and (**b**) distilled water.

The obtained calibration curves in tris-glycine and distilled water are given in Figure 1a,b.

### 2.5. Total Nitrogen by the Kjeldahl Method [28]

The determination of total nitrogen (N_t_) in a product of vegetable or animal origin can be done only after its mineralization by wetting. Wet mineralization, by boiling the product in concentrated sulfuric acid and in the presence of an oxidizing agent (perchloric acid, hydrogen peroxide or solid catalyst) releases nitrogen from the organic compounds present in the material under analysis and its fixation in the form of ammonium borate. Thus, transformed nitrogen can be determined either by a volumetric method (Kjeldahl method) or using a spectrophotometric method (the Nessler reaction). To determine N_t_ from ammonium borate (mineralization product), the ammonia presented in the solution is released by using sodium hydroxide and then it was distilled. In the obtained distillate, nitrogen was determined volumetrically using the titration method with a solution of hydrochloric acid of known concentration and a known factor in the presence of the Groack indicator (mixture of methyl red and methylene blue). The N_t_ from the sample is calculated from the amount of hydrochloric acid used in the titration.

0.5 g Glosa wheat and 0.5 g Amethyst barley cultivars were weighted. Also, 4 g disintegrant mixture consisting of 5 g of copper sulfate, 100 g potassium sulfate and 2.5 g selenium was weighted. The samples were placed in a Kjeldahl balloon. An amount of 10 mL concentrated sulfuric acid was added to the above mixture, and the beaker was connected to a digestion apparatus. The temperature was settled at 440 °C and it was kept in the flask for 30 min. At the end of the digestive stage, the contents of the Kjeldahl flask were quantitatively brought into the distillation flask by multiple washes with distilled water. By connecting the flask to the distillation plant, a 50 mL sodium hydroxide (30% wt.) was introduced into the solution. It was brought to half with distilled water. An amount of 50 mL 4% boric acid and 2–3 drops of Groack indicator solution (a mixture of methyl red and methylene blue indicator) were brought into the collection flask. The mixture was refluxed for 20–30 min or until the volume in the distillation flask reached 100 mL. Nitrogen was massively distilled in the first 5–6 min from the beginning of reflux. The contents of the distillation flask were titrated with a 0.1 N hydrochloric acid solution of known factor (0.9740), the volume of used acid was noted, and the N_t_ content of the sample and the total proteins using the conversion factor of 6.25 were calculated.

### 2.6. GC-MS/MS Analysis of Amino Acids Derived from Proteins

An amount of 100 mg wheat and 100 mg barley cultivars were weighed using the analytical balance into centrifuge tubes. The samples were made in duplicate. Half of the samples were brought to 5 mL with tris-glycine buffer and half with distilled water. The samples were centrifugated for 30 min, on ice, at 4000 rpm.

A 600 µL sample of 20 mg/mL was taken from each test tube and was then brought into derivatization vials and supplemented with 720 µL concentrated hydrochloric acid. The vials were stapled and were left in the oven for 8 h at 110 °C for protein hydrolysis process. Then, 250 µL was transferred to other vials and dried at 40–50 °C. After drying, the samples were brought with 250 µL acetonitrile, and were derivatized with 250 µL BSTFA and they were sealed.

The vials were introduced in the stirring module of the Gas Chromatograph coupled with the Quadrupol Triple Mass Spectrometer for one hour at a maximum temperature of 200 °C and a stationary one of 105 °C; afterward, the contents were transferred into injection vials and left for two hours. A standard solution of marine fish extract (Biotehnos S.A., Otopeni, Romania) in concentrations of 25 µL, 50 µL, 100 µL, 200 µL, and 400 µL was used to draw the calibration curve.

### 2.7. Protein Analysis by Polyacrylamide Gel Electrophoresis

The protein fingerprint of the samples is based on the separation of the polypeptide bands according to the molecular weight and after the migration into the polyacrylamide gel. The highlighting of the bands occurs by staining with reagents, which bind stoichiometrically, non-specifically, to the protein. The evaluation of their molecular weight was performed by comparison with a protein marker (20–220 kDa). The general principle of the method is described in the European Pharmacopoeia [29]. 

Reagents used: (A)—Samples from Tris-glycine buffer and distilled water previously obtained; (B)—Sample solubilization solution: 2.5 mL concentration gel buffer, 2.0 mL glycerol, 4 mL 10% SDS, 0.5 mL Coomassie Brilliant Blue G-250 0.1%, 1 mL purified water. To 950 µL solution for solubilization was added 50 mL samples; (C)—Acrylamide-bisacrylamide solution: 29.2 g acrylamide and 0.8 g N′N′—bis-methylene—acrylamide dissolved in 100 mL purified water; (D)—10% SDS solution: 10 g SDS is dissolved in 90 mL purified water with stirring and brought to a final volume of 100 mL with the same solvent; (E)—Gel buffer for separation: 18.15 g 1.5 M Tris-Hcl, pH = 8.8 Trizma base^®^ dissolved in 80 mL purified water. The pH was adjusted to 8.8 with 6 N HCl and it was brought to 100 mL with the same solvent; (F)—Concentration gel buffer: 6 g Tris-HCl (0.5 M), pH = 6.8 Trizma base^®^ was dissolved in 60 mL purified water. The pH to 6.8 was adjusted with 6 N HCl and brought to 100 mL with the same solvent; (G)—10% ammonium persulfate (APS): 0.1 g APS was dissolved in 1 mL purified water; (H)—TEMED: Tetramethylenediamine; the migration buffer pH = 8.3: it was weighed exactly 3.03 g Trizma base^®^, 14.400 g Glycine, 10 mL 10% SDS and it was brought to a final volume of 1000 mL. The pH was not adjusted. (I)—Fixation solution: Trichloroacetic acid (TCA) 12%; (J)—Staining solution: This was dissolved 0.18 g Coomassie Brilliant Blue G-250^®^ in 45 mL methanol, 45 mL purified water and 10 mL acetic acid; (K)—Discoloration solution: methanol: acetic acid: purified water in a ratio of 40:10:50 (*v*/*v*); (L)—Discoloration stop solution: 2% acetic acid in purified water; (M)—Tris-glycine buffer pH 8.3: This was a precisely weighed 0.6 g Trizma base^®^ and 2.88 g Glycine which were dissolved in purified water. It was brought to 100 mL with the same solvent. *Procedure*: The test solution is prepared from 60 µL sample solution (A) and 60 µL sample solubilization solution (B). It was stirred and kept in the water bath at 95 °C for 5 min.

The presence of proteins was highlighted by the appearance of distinct blue horizontal bands, the size of the molecular masses was assessed by comparison with the marker bands. The migration distances for the constituent polypeptides from the marker (from the starting line of the separation gel to the center of the band) and the total migration distance (from the starting line of the separation gel to the marked level of the dye front) were measured using the software Chemidoc. The graph was obtained by plotting log (M_w_) as a function of R_f_, where: M_w_ is the molecular mass of each protein in the marker mixture and R_f_ is the individual protein band migration distance/total migration front distance. Due to the low intensity of the bands obtained by staining with Coomasie Brilliant Blue, it was opted for a double marking with Thermo Scientific Pierce Silver Stain^®^ according to the following steps: The polyacrylamide gel was washed with water twice for 5 min and then the water was removed; A EtOH:Acetic Acid:Water = 3:1:6 was used twice for 15 min for the solution used for fixation; this was washed with 10% ethanol solution twice for 5 min, with water twice for 5 min and then the water was removed; the gel was immersed in a mixture of 25 mL water and 50 µL Silver Stain Sensitizer^®^ for 1 min; it was then washed with water twice for 1 min and the water was then removed; 25 mL water and 250 µL Silver Stain Enhancer^®^ were immersed in the mixture for 5 min; it was washed with water twice for 20 s and the water was removed; 25 mL Silver Stain Developer^®^ and 250 µL Silver Stain Enhancer^®^, were mixed for 30 s until the strips became visible; the reaction was stopped with a solution of 5% acetic acid.

### 2.8. Dosing of Fructan Fibers Using the Seliwanoff Method

The Seliwanoff method is based on the differentiation between ketosis and aldoses. At high temperatures, ketoses are hydrolyzed faster than aldoses, with the breakdown of the carbohydrate cycle and with the formation of furfural. This reacts in an acidic medium with the resorcinol solution resulting in a red color that can be measured spectrophotometrically at a wavelength of 520 nm.

**Figure 2:**
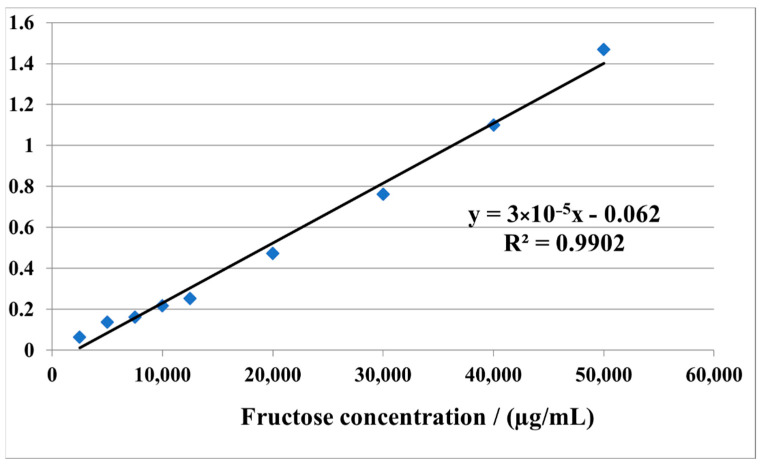
The calibration curve for dosing of fructans.

Fructose stock solution of 1 mg/mL, 1% resorcinol solution (1 g resorcinol, 0.25 g thiourea, 100 mL glacial acetic acid) and 5:1 hydrochloric acid solution was prepared. Nine successive dilutions were prepared from the stock solution for drawing the calibration line by taking 25 µL, 50 µL, 75 µL, 100 µL, 125 µL, 200 µL, 300 µL, 400 µL, 500 µL. The standard solutions and 500 µL sample were treated with 0.5 mL distilled water, 0.5 mL 1% resorcinol solution and 3.5 mL hydrochloric acid solution. The tubes were heated in a water bath at 80 °C for 10 min, then they were cooled. They were then read on the Perkin Elmer Life and Analytical Science LAMBDA 25 molecular absorption spectrophotometer at 520 nm in a maximum of 30 min. The calibration curve is presented in Figure 2.

All analyses were effectuated in triplicate, and the data were registered as means values ± standard deviation (SD).

### 2.9. Measurements

The absorbances of the samples were obtained using a Perkin Elmer Life and Analytical Science Molecular Absorption Spectrophotometer LAMBDA 25, at a λ = 595 nm. For the total nitrogen determination by the Kjeldahl method, a Digesdahl 23130–21 digestion apparatus was used. A Thermo Scientific TSQ 8000 EVO gas chromatograph was used under the following conditions: Column, Thermo Scientific TraceGOLD TG-5SilMS, L = 30.00 m, D = 0.25 mm; The used carrier gas, He; Flow rate, 1 mL/min; Analysis time, 46.667 min; Detection with Total Ion Chromatogram (ICT), 40–600 *M/z*; Software, Chromeleon 7. Protein analysis by polyacrylamide gel electrophoresis was performed using the Bio-Rad PowerPac Basic Mini electrophoresis system. A Perkin Elmer Life and Analytical Science LAMBDA 25 molecular absorption spectrophotometer was used for dosing of fructan fibers at 520 nm.

## 3. Results

### Qualitative Assessment of Wheat Cultivars and Barley Cultivars

Due to the increasing use of gluten-free products for different food applications [30], it is necessary to achieve deep characterization of the content of gluten in the studied cultivars and to determine whether differences in composition are dependent from the cereal cultivars type. The results of gluten detection from wheat are presented in Table 3.

According to the gluten concentrations in the flour, they can be divided into: (i) low gluten flour (7–8%); (ii) usual flour (8–12%); (iii) flour with high gluten content (12–13%); (iv) gluten-free flour [31]. The dosage of gluten from the five wheat samples, as can be seen in Table 3, highlighted the presence of gluten quantities greater than 13%. 

The protein concentrations of the five selected wheat cultivars and five barley cultivars were determined using the extraction from tris-glycine buffer and distilled water. The results are presented in Table 4 and Table 5.

The experimental results regarding the concentration of proteins for Glosa wheat and Ametist barley cultivars obtained using the Kjeldahl method are shown in Table 6. 

Considered as a reference method for the dosing of proteins from plant and animal products, the Kjeldahl method was further used to extrapolate the results obtained previously using the Bradford method. The results are presented in Table 7.

The experimental results, obtained after extrapolation from Table 7, indicated an increase or a very high content in the case of wheat samples of proteins. This increase in protein concentration can be justified by the increase in gluten content. 

The calculated amino acid concentrations from studied cultivars based on peak areas obtained using Chromeleon 7 software from GS-MS/MS analysis are represented in Table 8.

**Figure 3:**
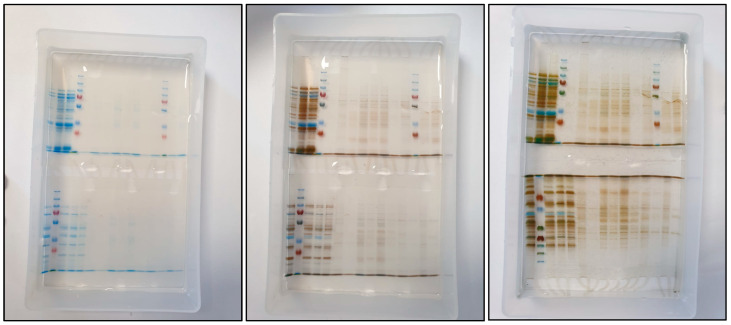
Polyacrylamide Gel Staining Steps Using Coomassie Brilliant Blue G-250^®^ and Thermo Scientific Pierce Silver Stain^®^ Dye.

**Figure 4:**
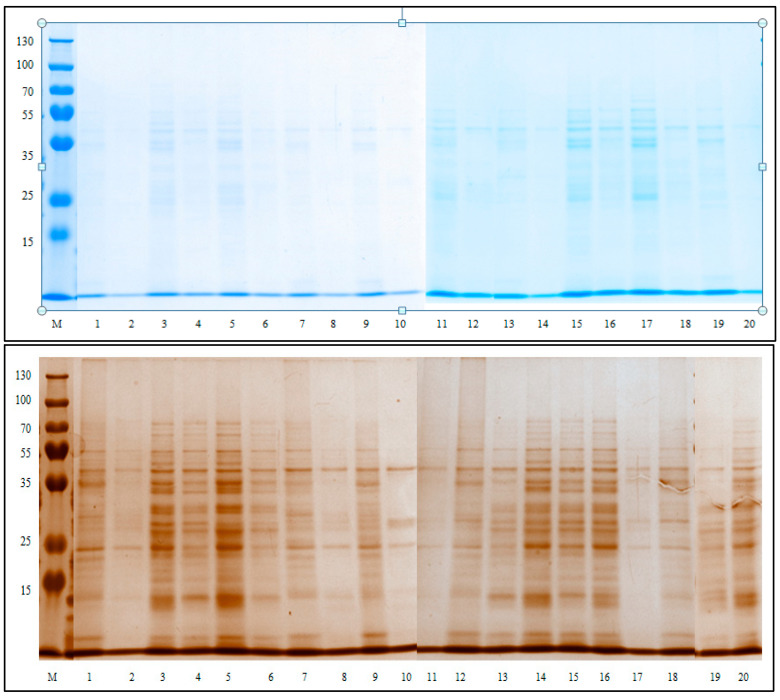
Polyacrylamide gels after migration of colored samples with Coomassie Brilliant Blue G-250^®^ and Thermo Scientific Pierce Silver Stain^®^ (M-Thermo molecular marker (kDa); 1—Simbol in tris-glycine; 2—Simbol in distilled water; 3—Pitar in tris-glycine; 4—Pitar in distilled water; 5—Pajura in tris-glycine; 6—Pajura in distilled water; 7—Onix in tris-glycine; 8—Onix in distilled water; 9—Ametist in tris-glycine; 10—Ametist in distilled water; 11—Litera in tris-glycine; 12—Litera in distilled water; 13—Smarald in tris-glycine; 14—Smarald in distilled water; 15—Dropia in tris-glycine; 16—Dropia in distilled water; 17—Glosa in tris-glycine; 18—Glosa in distilled water; 19—Dana in tris-glycine; 20—Dana in distilled water.).

The results of protein analysis by polyacrylamide gel electrophoresis are shown in photographs from Figure 3 and Figure 4.

The strips from the two sample gels were scanned and were identified using the Chemidoc program. Subsequently, it was decided to process the data corresponding to the double-labeled gels, stained with Thermo Scientific Pierce Silver Stain reagent. Comparing the samples extracted in tris-glycine buffer with those extracted in water, a high extraction yield can be observed in the case of the buffer solution. The bands have been associated with proteins type based on literature data. In the case of both wheat and barley cultivars, important proteins have been successfully identified and included in their mass domains. The results are represented in Table 9 and Table 10.

The results of fructan dosage are shown in Table 11.

It can be seen that, in most cases, the extraction in distilled water had a better yield.

## 4. Discussions

Gluten remains an essential protein for the food and bakery industry. Given that this quantitative analysis was performed on raw material, the impact of crossing and selection methods of improvement on the gluten content of the wheat samples was demonstrated. The literature supports the fact that the proteins (gluten) from wheat, which are associated with the elasticity and extensibility of the bakery and related products, play an important role in human nutrition [32]. Gluten represents one of the major protein components of wheat (~80% from the total protein endosperm) in which gliadins and glutenins represent the most important parts. These two types of gluten determine its quality by their interaction and variation in composition [33,34,35]. The highest increase in the gluten content (Table 3) was observed in the case of Pitar wheat, where the amount of 19.60 ± 0.20% is 6.5% higher than in the case of commercial flour with a high gluten content. In the case of the other cultivars, the increase was not so pronounced. An increase of 3.4%in the maximum gluten content was observed in the case of the Dropia cultivars (16.46 ± 0.16%).

The literature data report that the quality of the final products is affected by the flour protein content. The protein is represented by the different proportions of low-molecular-weight glutenin subunit (LMW-GS), high-molecular-weight glutenin subunit (HMW-GS), albumins, α-, γ-, and ω-gliadins, and globulins [36,37,38,39]. According to the Department of Agriculture of the United States of America (USDA), the protein content in wheat flour is between 6.67 and 11.8%, and the protein content in barley is between 9 and 10% [40]. The results obtained using the Bradford assay (Table 4 and Table 5) were below the lower limit. This is probably due to the inefficiency of protein extraction using a single solvent (tris-glycine buffer or distilled water) and the structural differences between plant and animal proteins used as standard Bovine Serum Albumin (BSA) in this dosage. Subsequently, for developing a future quantitative extraction method, the total nitrogen (N_t_) was dosed using the Kjeldahl method.

The results of the GC-MS/MS analysis (Table 8) showed quantitative and qualitative differences between different cultivars from the same species. For example, the amino acid L-proline was not identified in the Pajura and Dropia wheat cultivars, but occurs in the other three, and Tyrosine is absent only in the case of Dropia wheat. From a quantitative perspective, an increased content of L-Glutamic acid (0.68–1.94%) and DL-Phenylalanine (0.1–0.53%) can be observed in all wheat samples. In the case of other amino acids, no significant differences were observed between cultivars or important concentrations. However, the Glosa wheat cultivars proved to be clearly superior in terms of the concentration of each amino acid.

Qualitative differences in barley cultivars showed a lack of L-Isoleucine in Onix, L-Proline in Symbol and Onix and Tyrosine in Dana, Symbol and Onix. The glutamic acid content was increased compared with other amino acids present in the sample (0.14–1.04%). The concentrations of the other amino acids were compared, but there were no major differences. No quantitatively superior cereal cultivars were found. The plant-based proteins have relatively low essential amino acid contents when compared with animal-based and human proteins. This assumption was demonstrated by comparing the amino acids content of various plants and animal-based protein sources [41,42].

According to the literature, in the case of wheat, non-gluten proteins represent 20–25% of the total mass of proteins, most of which are monomeric [43]. Albumins and globulins represent about 14% and 22%, respectively. Molecular weights are generally of 25 kDa, although there are significant proportions between 60 and 70 kDa. From the nutritional perspective, these proteins have a balanced intake of amino acids (high content of lysine, tryptophan, methionine). Wheat gluten can be separated into three broad groups: sulfur-rich proteins of about 50 kDa (α-, β-, γ-gliadins and B-, C-glutenins), low-sulfur proteins of the same mass (ω-gliadins and D-glutenins) and high mass proteins of about 100 kDa. Glutenins and gliadin make up 60–90% of all wheat proteins and tend to be rich in asparagine, glutamine, arginine or proline, but poor in nutritionally important amino acids such as lysine, tryptophan or methionine [44].

Proteins separated from barley cultivars can be classified into several fractions: albumin, globulin, prolamine (hordein) and gluteline. These represent 8–15% of the seed mass, of which 3–5% are albumin and 10–20% globulin. The main components are hordein and glutelin, representing 40–45% of the total mass. According to the literature, three protein bands were highlighted: BPI-1 (albumin and globulin fraction with masses less than 20 and between 30 and 70 kDa), and BPI-2 and BPI-3 (C-, D-, B- hordein with masses between 29 and 55 kDa) [45]. Previous studies have shown that fructans, soluble fiber, are good for human health [46]. Fructans are non-reducing polysaccharides composed of fructose units and terminated with a glucose molecule, which have different degrees of polymerization [46]. In wheat, fructans have a value of 0.7–2.9%, and in barley 0.9–4.5%. This concentration is high enough to regulate intestinal transit and acts as a probiotic in the intestine. However, in some cases it can cause symptoms such as abdominal pain, flatulence, diarrhea, constipation, and intestinal cramps, both in patients with irritable bowel syndrome and in non-celiac patients [47]. Experimentally obtained concentrations ranged from 1.01 ± 0.04 to 2.27 ± 0.24% fructose for wheat cultivars and from 1.28 ± 0.22% to 1.75 ± 0.16% for barley cultivars. Comparing the results obtained after the fructose dosing process, it was observed that some cultivars of the same species have double saccharide concentrations compared to others, examples being wheat Dropia in the first case, and barley Onix cultivars in the second one.

All the above analyzed cultivars have concentrations consistent with the data found in the literature, but the differences that appear compared to them can be based on the used breeding techniques. Thus, future studies can be performed to improve or decrease the fructan content of the desired cultivars.

Bread and bakery products are food products that occupy a high percentage in the food preferences of Romanians, as evidenced by recent studies conducted both during the pandemic and before [48,49]. As a result, the development of cereal cultivars with good baking power is an important aspect of the bakery industry. This involves an increase in the amount of gluten and in fermentable carbohydrates. Moreover, the increase in the amount of protein and especially in the amino acid content improves the nutritional value of bakery products. Moreover, fructan enrichment can have positive effects on bakery products, due to the increase in prebiotic content, fructan behaving as a fiber with beneficial effects on the intestinal microbiome and at the same time affecting the stimulation of the intestinal transit, thus helping to detoxify the body [50].

Several scientific studies have demonstrated the importance of using improved cultivars of wheat in obtaining bakery products with superior digestibility and special aroma [51,52,53].

Nevertheless, we must also consider numerous restrictive aspects, the varieties that are enriched in gluten and fructan are not recommended for the manufacture of bakery products for people who have an intolerance to them or celiac disease.

If wheat cultivars obtained with improved characteristics for the bakery industry are a valuable raw material for obtaining quality products, then barley cultivars with higher concentrations of valuable nutrients are important raw materials for the beer industry. Numerous studies carried out on different varieties of beer have demonstrated that the organoleptic properties of beer and its nutritional value are strongly influenced by the quality of the raw materials and the characteristics of the technological process. In this sense, the use of improved cultivars of barley leads to the obtaining of quality malt that, through their content of carbohydrates, vitamins, mineral salts, fibers, and proteins with increased biological value, helps to create nutritional, aromatic drinks with optimal alcohol content [54,55,56].

In addition to the process of obtaining improved cultivars, the qualities of the cultivation areas must be considered because environmental pollution or the practice of intensive agriculture can lead to the accumulation of toxic substances in the cereal grains, affecting the quality of the raw material and the safety of consumption [57,58,59,60].

## 5. Conclusions

In conclusion, this study has shown the quantity and quality differences in terms of gluten, protein, amino acids and sugar parameters of both wheat cultivars of *Triticum aestivum* L. and barley cultivars *Hordeum vulgare* L., respectively. Multiple studies have been conducted based on conventional dosing methods (gluten dosing), spectrophotometric (Bradford and protein dosing), digestion (Kjeldahl protein dosing), gas chromatography (GC- MS amino acid from protein dosing), or qualitative methods (identification of protein fractions by polyacrylamide gel electrophoretic method). Large differences in gluten concentration, protein, amino acids and carbohydrates were noted for both cultivars. These types of studies regarding the new wheat and barley cultivars are the maximum exploitation of interventions based on crossing and selection methods, beyond those which acquired a better yield. Changing the nutritional profile by improving and dosing the constituent nutrients can lead to the emergence of special diets for patients over time, based on their needs or deficiencies. Obtaining wheat and barley cultivars with high concentrations of valuable nutrients (especially protein and fructan) represents a success and improvement for both the bakery and beer industries because the use of fortified raw materials does not rise to the level of quality of improved natural products. The greatest applicability for the bakery and beer industries was determined for all of the studied wheat and barley cultivars due to the high values of their nutritional constituents, which may contribute to increased interest in this cultivar by bakery and beer producers. This study highlighted the production of improved cereal cultivars, with increased content of the main nutrients involved in the quality of food products derived from them. The organoleptic characteristics and nutritional value of bakery products and beer varieties obtained from these cultivars will be the future scope of the study.

## Figures and Tables

**Table 1:** The morphological and physiological characteristics of the wheat cultivars (*Triticum aestivum* L.) and barley cultivars (*Hordeum vulgare* L.) [26].

Cereal Cultivars	Morphological Characteristics	Physiological Characteristics	Cereal Quality
The wheat cultivars			
Glosa	It has a semi-erect plant twig, in the twinning phase. The leaf has a semi-bent bearing after the flowering phase. The average height of the plant is about 85–95 cm. The grain-bearing tip part of the stem is white, aristate, cylindrical in shape and it has a medium density. The grains are of medium size, elongated in shape. The color is red, and, under normal cultivation conditions, they have a mass of 42–43 g/1000 grains and a mass of 76–82 kg/hL;	It is an early cultivar type with a good resistance to fall, to winter, the drought and the heat and has also a good resistance to sprouting. It has a medium resistance to brown rust, and it is resistant to powdery mildew and current yellow rust strains;	It is characterized by hard gluten. It has average protein content and bread volume.
Dropia	It has a semi-erect plant bush in the twinning phase. The leaf has a semi-erect bearing after the flowering phase. The average height of the plant is 85–95 cm. The grain-bearing tip part of the stem is white, fusiform, large and of medium density. The grains are of medium size, elongated in shape, have a red color and, under normal growing conditions, a mass of 41–46 g/1000 grains and a mass of 76–80 kg/hL;	It is an early cultivar type with a medium resistance to falling. It is resistant to winter, drought and heat. This cultivar is medium sensitive to brown rust, yellow rust and powdery mildew;	It proved to have good milling and baking indices. In general, this cultivar is characterized by a good consistency of bakery quality.
Pitar	It has a semi-erect plant bush, in the twinning phase; the leaf has a semi-erect bearing after flowering. The leaves are medium in length and width, and they are covered with a light waxy layer. The average height of the plant is 80–95 cm. The spike is white, of medium density, aristate, pyramidal in shape, of medium length and with a semi-nuanced position at maturity. The grains are medium-sized, well-filled, elongated, and red in color. Mass of 1000 grains: 42–44 g. Hectoliter mass: 79–82 kg/hL;	It has good resistance to falling, wintering, drought and heat. It is resistant to brown rust and powdery mildew and medium resistant to Septoria and yellow rust. It has a medium level of resistance to ear Fusariosis.	It is distinguished by very good bakery quality, being from this point of view superior to the Glosa cultivars.
Pajura	It has a semi-erect plant bush in the twinning phase. The leaves are medium in length and width, and they are covered with an intense waxy layer and the average height of the plant is between 75 and 92 cm, being shorter than the control cultivars Dropia and Glosa by 5–10 cm. The grains are of medium size, elongated shape, red in color and have, under normal cultivation conditions, a mass of 40–44 g/1000 grains and a hectoliter mass of 77–80 kg/hL;	The cultivar is early type (with a vegetation period similar to the control cultivars Dropia and Glosa), with very good resistance to falling, good resistance to wintering, drought and heat. Pajura wheat cultivar is resistant to brown rust and powdery mildew and medium resistant to Septoria and yellow rust, and it has a medium level of resistance to fusariosis;	It has a good baking quality, being from this viewpoint similar to the Glosa cultivar. It achieves on average production an increase of 5–8% compared to the Glosa cultivars, in the same technological conditions, the increases being higher in favorable conditions of attack of foliar diseases.
Litera	It has a semi-lying to lying plant bush, in the twinning phase. The leaves have a semi-bent position after the flowering phase. The leaves are medium in length and width, and they are covered with a not too intense waxy layer in the second part of the grain filling period. The average height of the plant is between 95 and 105 cm, being the same height as the control cultivars Dropia. The spike is white, semi-dense, aristate, pyramidal in shape and with a semi-nuanced position at maturity. The grains are of medium size, elongated in shape, have a red color and have, under normal cultivation conditions, a mass of 1000 grains of 42–45 g and a hectoliter mass of 77–81 kg/hL;	Early cultivars, with a vegetation period similar to the control cultivars Dropia, with good resistance to fall, winter, drought and heat. It is resistant to brown rust and medium resistant to current breeds of yellow rust and powdery mildew. It has a medium level of resistance to fusariosis.	Tested both on laboratory microplates and in the pilot phase, the Litera cultivars proved to have good baking characteristics. The quality indices of these cultivars are quite close to those of the Dropia cultivars.
The barley cultivars			
Dana	The reference cultivars		
Simbol	Typical semi-early autumn cultivars, with a good twinning capacity, medium to high waist, with medium length spike, yellow color and long edges; the mass of 1000 grains varied between 40 and 45 g, the average protein content was 10.5–12.5%, the average starch content was 62.0% (qualitative parameters depend on the technology applied but also on the conditions climate change).	It has superior resistance to winter, fall and foliar (environment resistant to brown reticular staining of barley leaves *(Pyrenophora teres f. teres*);	It achieved a 17% increase in production compared with the control cultivars Dana under the same technological conditions
Ametist	Typical semi-early autumn cultivars, with good twinning capacity, medium size, with the spike of medium length, yellow color and long edges; The mass of 1000 grains varied between 44 and 52 g, the average protein content is 9.9–12.3%, the average starch content is 57.9–64.4%	It has superior resistance to winter, fall and leaf, environmental resistant to the brown reticular staining of barley leaves (*Pyrenophora teres f. teres*);	The assortment level (I + II) can reach a maximum of 94.5% (qualitative parameters depend on the applied technology but also on the climatic conditions)
Onix	It is a genotype of autumn barley, with a growing period equal to that of the control cultivars Dana. It has a good twinning capacity, medium to high waist, with medium length spike, and long anthocyanin-colored edges. The mass of 1000 grains had values between 46.0–50.0 g, the protein content was 10.4–11.9%, and the starch content was 62.3%.	The cultivars are semi-early, uniform, with superior resistance to wintering, fall, foliar and ear diseases (environment resistant to brown reticular staining of barley leaves-*Pyrenophora teres f. teres*)	Qualitative parameters depend on the applied technology, but also on the climatic conditions.
Smarald	The shape is intermediate, with a medium to high frequency of plants, with a curved flag leaf. Plant height is medium.	Semi-early cultivars, with good resistance to wintering, falling and foliar diseases.	Its basic characteristic is the high productivity and the quality of the grains.

**Table 2:** The used concentrations for the calibration curves.

BSA Concentration	BSA Volume (1 mg/mL)	Tris-Glycine/Distilled Water
0.05	5 µL	95 µL
0.1	10 µL	90 µL
0.2	20 µL	80 µL
0.4	40 µL	60 µL
0.6	60 µL	40 µL

**Table 3:** Measured contents of gluten in wheat cultivars (means values ± standard deviation).

The Wheat Cultivars	Initial Mass (g)	Wet Mass (g)	Dry Mass (g)	Gluten Concentration (%)
Glosa	10.01 ± 0.02	3.67 ± 0.03	1.32 ± 0.02	13.22 ± 0.12
Dropia	10.00 ± 0.12	4.52 ± 0.05	1.65 ± 0.06	16.46 ± 0.16
Pitar	10.07 ± 0.06	5.31 ± 0.02	1.96 ± 0.11	19.60 ± 0.20
Pajura	10.01 ± 0.04	4.74 ± 0.04	1.63 ± 0.09	16.32 ± 0.13
Litera	10.01 ± 0.08	4.18 ± 0.06	1.56 ± 0.08	15.60 ± 0.11

**Table 4:** Measured contents of the extracted proteins concentration in tris-glycine buffer (means values ± standard deviation).

Sample	Used Volume (µL)	Absorbance	Sample Concentration (µg/mL)	Sample Mass (mg)	Protein Concentration (%)
Wheat cultivars					
Pajura	50	0.275	185.9	50.0	3.72 ± 0.14
Litera	50	0.285	192.8	53.2	3.62 ± 0.03
Pitar	50	0.305	206.1	50.5	4.08 ± 0.06
Dropia	50	0.313	211.7	51.4	4.12 ± 0.07
Glosa	50	0.293	198.3	52.6	3.77 ± 0.05
Barley cultivars					
Dana	50	0.237	159.5	50.5	3.16 ± 0.03
Simbol	50	0.208	139.7	53.6	2.61 ± 0.02
Ametist	50	0.251	169.2	54.0	3.13 ± 0.06
Onix	50	0.243	163.7	52.6	3.11 ± 0.14
Smarald	50	0.189	126.6	54.4	2.33 ± 0.11

**Table 5:** Measured contents of the extracted proteins concentration in distilled water (means values ± standard deviation).

Sample	Used Volume (µL)	Absorbance	Sample Concentration (µg/mL)	Sample Mass (mg)	Protein Concentration (%)
Wheat cultivars					
Pajura	50	0.114	82.19 ± 0.22	53.2	1.55 ± 0.14
Litera	50	0.117	84.29 ± 0.16	56.4	1.49 ± 0.06
Pitar	50	0.126	90.44 ± 0.23	54.6	1.66 ± 0.14
Dropia	50	0.194	137.90 ± 0.32	57.9	2.38 ± 0.08
Glosa	50	0.107	76.95 ± 0.12	56.7	1.36 ± 0.12
Barley cultivars					
Dana	50	0.149	106.90 ± 0.18	55.6	0.96 ± 0.11
Simbol	50	0.134	96.16 ± 0.12	55.8	0.86 ± 0.12
Ametist	50	0.145	103.60 ± 0.22	54.2	0.96 ± 0.12
Onix	50	0.158	112.90 ± 0.24	56.4	1.00 ± 0.08
Smarald	50	0.159	113.70 ± 0.20	52.6	1.08 ± 0.13

**Table 6:** Measured contents of the protein concentrations obtained using the Kjeldahl method (means values ± standard deviation).

Cereal Cultivars	Mass (g)	HCl (mL)	N_t_ (%)	Proteins (%)
Glosa wheat	0.52 ± 0.02	7.8	2.04 ± 0.04	12.80 ± 0.06
Ametist barley	0.50 ± 0.02	5.9	1.59 ± 0.02	9.99 ± 0.04

**Table 7:** The results obtained using the extrapolation method (means values ± standard deviation).

Cereal Cultivars	Proteins by Bradford (%)	Calculated Conversion Factor	Total Proteins (%)
Wheat cultivars			
Pajura	1.54 ± 0.04	9.48	14.59 ± 0.11
Litera	1.49 ± 0.13	9.48	14.12 ± 0.22
Pitar	1.65 ± 0.05	9.48	15.64 ± 0.06
Dropia	2.38 ± 0.08	9.48	22.56 ± 0.08
Glosa	1.35 ± 0.12	9.48	12.79 ± 0.02
Barley cultivars			
Dana	0.96 ± 0.04	10.51	10.08 ± 0.06
Simbol	0.86 ± 0.02	10.51	9.03 ± 0.04
Ametist	0.95 ± 0.11	10.51	9.98 ± 0.15
Onix	1.00 ± 0.14	10.51	10.51 ± 0.06
Smarald	1.08 ± 0.06	10.51	11.35 ± 0.08

**Table 8:** Measured contents of the amino acid concentrations (means values ± standard deviation).

	Concentration of Compound (%)									
Wheat Cultivars						Barley Cultivars				
Compound	Pajura	Litera	Pitar	Dropia	Glosa	Dana	Simbol	Ametist	Onix	Smarald
L-Valine	0.19 ± 0.08	0.15 ± 0.06	0.25 ± 0.04	0.14 ± 0.02	0.31 ± 0.03	0.07 ± 0.11	0.06 ± 0.31	0.09 ± 0.02	0.04 ± 0.14	0.06 ± 0.31
Glicine	17.54 ± 0.11	18.09 ± 0.32	21.42 ± 0.04	18.06 ± 0.02	25.81 ± 0.08	22.28 ± 0.04	24.99 ± 0.06	26.96 ± 0.16	13.93 ± 0.33	25.25 ± 0.26
L-Leucine	0.12 ± 0.07	0.09 ± 0.05	0.13 ± 0.02	0.08 ± 0.08	0.16 ± 0.13	0.08 ± 0.02	0.06 ± 0.01	0.10 ± 0.04	0.04 ± 0.03	0.07 ± 0.11
L-Isoleucine	0.13 ± 0.04	0.07 ± 0.01	0.12 ± 0.03	0.07 ± 0.01	0.17 ± 0.04	0.07 ± 0.03	0.06 ± 0.04	0.11± 0.08	-	0.06 ± 0.03
L-Serine	0.08 ± 0.01	0.08 ± 0.01	0.10 ± 0.02	0.07 ± 0.01	0.13 ± 0.02	0.06 ± 0.01	0.05 ± 0.01	0.07 ± 0.04	0.03 ± 0.01	0.05 ± 0.11
L-Threonine	0.06 ± 0.01	0.05 ± 0.01	0.07 ± 0.01	0.05 ± 0.01	0.18 ± 0.04	0.08 ± 0.01	0.05 ± 0.01	0.11 ± 0.02	0.03 ± 0.01	0.05 ± 0.01
L-Proline	-	0.03 ± 0.01	0.19 ± 0.02	−	0.38 ± 0.06	0.27 ± 0.04	-	0.65 ± 0.06	-	0.17 ± 0.12
L-Aspartic acid	0.17 ± 0.12	0.18 ± 0.14	0.24 ± 0.13	0.12 ± 0.11	0.31 ± 0.24	0.16 ± 0.22	0.14 ± 0.12	0.23 ± 0.14	0.06 ± 0.01	0.17 ± 0.14
L-Glutamic acid	1.24 ± 0.08	1.01 ± 0.12	1.45 ± 0.23	0.69 ± 0.03	1.95 ± 0.04	0.56 ± 0.07	0.43 ± 0.02	1.05 ± 0.06	0.15 ± 0.01	0.54 ± 0.14
DL-Phenylalanine	0.29 ± 0.17	0.22 ± 0.32	0.29 ± 0.15	0.11± 0.11	0.53 ± 0.03	0.11 ± 0.08	0.25 ± 0.02	0.23 ± 0.12	0.02 ± 0.21	0.14 ± 0.26
Tyrosine	0.04 ± 0.04	0.02 ± 0.08	0.04 ± 0.06	-	0.07 ± 0.01	-	-	0.04 ± 0.11	-	0.03 ± 0.21

**Table 9:** Identification of proteins from the colored sample strips using Thermo Scientific Pierce Silver Stain^®^ (Wheat cultivars).

Mass (kDa)	Types of the Proteins	Wheat Cultivars in Tris-glycine					Wheat Cultivars in Distilled Water				
		Pajura	Litera	Pitar	Dropia	Glosa	Pajura	Litera	Pitar	Dropia	Glosa
112.8	HMW glutenin	X		X			X		X	X	
108.1											
101.4		X		X	X		X		X	X	
96.2					X				X		
87.0		X		X	X		X	X	X	X	X
79.9											
77.0		X		X	X				X	X	
72.6	Ω-gliadin, LMW glutenin	X	X	X	X	X	X	X	X	X	X
63.9		X		X	X		X	X	X	X	X
60.0		X		X	X		X		X	X	X
51.7	γ-gliadin, LMW glutenin	X		X			X		X	X	
47.0					X						
45.0		X		X	X	X	X	X	X		X
42.2	α-gliadin, LMW glutenin									X	
40.5		X		X	X		X		X	X	
34.5		X	X	X	X	X	X	X	X	X	X
31.5		X		X	X			X	X	X	X
28.5											
25.8				X	X			X		X	X
22.0		X		X	X		X	X	X	X	X
19.0										X	
14.9		X	X	X	X		X	X	X	X	X

**Table 10:** Identification of proteins from the colored sample strips using Thermo Scientific Pierce Silver Stain^®^ (Barley cultivars).

Mass (kDa)	Types of the Proteins	Barley Cultivars in Tris-glycine					Barley Cultivars in Distilled Water				
		Dana	Simbol	Ametist	Onix	Smarald	Dana	Simbol	Ametist	Onix	Smarald
112.8	D-hordein				X						X
108.1					X						
101.4							X				X
96.2			X								X
87.0			X	X	X	X	X	X			X
79.9			X		X		X				
77.0			X								X
72.6	C-hordein	X	X	X	X	X		X	X	X	X
63.9			X	X	X	X					X
60.0											X
51.7		X	X	X	X						X
47.0			X	X	X			X			
45.0						X		X	X		X
42.2	B-hordein	X		X				X			
40.5		X		X		X					X
34.5		X	X	X	X	X		X	X	X	X
31.5			X	X	X	X					
28.5			X	X		X					
25.8	Albumin	X									
22.0		X	X	X	X	X		X		X	X
19.0	Globulin				X					X	
14.9		X	X	X	X	X					X

**Table 11:** Fructan dosage in cereal cultivars (means values ± standard deviation).

Sample	Used Volume (µL)	Absorbance	Sample Concentration (µg/mL)	Mass Sample (mg)	Fructose (%)
Wheat cultivars					
Pajura in Tris-glycine	500	0.229	9730.0	53.2	1.83 ± 0.14
Pajura in distilled water	500	0.274	1119.0	53.2	2.10 ± 0.16
Litera Tris-glycine	500	0.184	8190.0	56.4	1.45 ± 0.21
Litera in distilled water	500	0.162	7450.0	56.4	1.32 ± 0.22
Dropia Tris-glycine	500	0.233	9816.7	57.9	1.69 ± 0.13
Dropia in distilled water	500	0.331	1313.3	57.9	2.27 ± 0.24
Pitar Tris-glycine	500	0.188	8323.3	54.6	1.52 ± 0.13
Pitar in distilled water	500	0.105	5576.7	54.6	1.02 ± 0.12
Glosa Tris-glycine	500	0.171	7753.3	56.7	1.37 ± 0.16
Glosa in distilled water	500	0.172	7793.3	56.7	1.37 ± 0.08
Smarald Tris-glycine	500	0.216	9260	52.6	1.76 ± 0.07
Smarald in distilled water	500	0.081	4780.0	54.2	0.88 ± 0.12
Ametist Tris-glycine	500	0.102	5473.3	54.2	1.01 ± 0.04
Ametist in distilled water	500	0.122	6143.3	54.2	1.13 ± 0.05
Barley cultivars					
Dana Tris-glycine	500	0.181	8113.3	55.6	1.46 ± 0.04
Dana in distilled water	500	0.202	8796. 7	55.6	1.58 ± 0.05
Simbol Tris-glycine	500	0.152	7130.0	55.8	1.28 ± 0.22
Simbol in distilled water	500	0.207	8950.0	55.8	1.60 ± 0.05
Onix Tris-glycine	500	0.209	9060.0	56.4	1.61 ± 0.07
Onix in distilled water	500	0.235	9890.0	56.4	1.75 ± 0.16

## Data Availability

Not applicable.

## References

[1] Lafiandra D., Riccardi G., Shewry P.R. (2014). Improving cereal grain carbohydrates for diet and health. J. Cereal Sci..

[2] Khan J., Khan M.Z., Ma Y., Meng Y., Mushtaq A., Shen Q., Xue Y. (2022). Overview of the Composition of Whole Grains’ Phenolic Acids and Dietary Fibre and Their Effect on Chronic Non-Communicable Diseases. Int. J. Environ. Res. Public Health.

[3] Lattimer J.M., Haub M.D. (2010). Effects of Dietary Fiber and Its Components on Metabolic Health. Nutrients.

[4] Arslain K., Gustafson C.R., Rose D.J. (2021). The Effect of Health Prompts on Product Consideration, Attention to Information, and Choice in Large, Online Product Assortments: The Case of Fiber. Food Qual. Prefer..

[5] Arslain K., Gustafson C.R., Rose D.J. (2020). Point-of-Decision Prompts Increase Dietary Fiber Content of Consumers’ Food Choices in an Online Grocery Shopping Simulation. Nutrients.

[6] Kasi P.M., Shahjehan F., Cochuyt J.J., Li Z., Colibaseanu D.T., Merchea A. (2019). Rising Proportion of Young Individuals with Rectal and Colon Cancer. Clin. Colorectal Cancer.

[7] Macfarlane G.T., Macfarlane S. (2012). Bacteria, colonic fermentation and gastrointestinal health. J. AOAC Int..

[8] Andarwulan N., Kurniasih D., Apriady R.A., Rahmat H., Roto A.V., Bolling B.W. (2012). Polyphenols, carotenoids, and ascorbic acid in underutilized medicinal vegetables. J. Funct. Foods.

[9] Vignolini P., Urciuoli S., Heimler D., Romani A. (2018). Carotenoids, Polyphenols and Antioxidant Activity Evaluation in Stone-Grinded Wheat Semolina. J. Health Sci..

[10] Gürel F., Öztürk Z.N., Uçarlı C., Rosellini D. (2016). Barley Genes as Tools to Confer Abiotic Stress Tolerance in Crops. Front. Plant Sci..

[11] Baik B.K., Ullrich S.E. (2008). Barley for food: Characteristics, improvement, and renewed interest. J. Cereal Sci..

[12] Quinde-Axtell Z., Baik B.-K. (2006). Phenolic compounds of barley grain and their implication in food products discolouration. J. Agric. Food Chem..

[13] Quinde-Axtell Z., Powers P., Baik B.-K. (2006). Retardation of discolouration in barley flour gel and dough. Cereal Chem..

[14] Kumar A., Sharma M., Kumar S., Tyagi P., Wani S.H., Gajula M.N.V.P., Singh K.P. (2018). Functional and structural insights into candidate genes associated with nitrogen and phosphorus nutrition in wheat (*Triticum aestivum* L.). Int. J. Biol. Macromol..

[15] Petimar J., Moran A.J., Ramirez M., Block J.P. (2020). A Natural Experiment to Evaluate the Nutritional Content of Restaurant Meal Purchases after Calorie Labeling. J. Acad. Nutr. Diet..

[16] Hovanet M.V., Ancuceanu R., Dinu M., Oprea E., Budura E.A., Negreṣ S., Velescu B., Duṭu L., Anghel I., Ancu I. (2016). Toxicity and anti-inflammatory activity of *Ziziphus jujuba* Mill. Leaves. Farmacia.

[17] Paduraru D.N., Coman F., Ozon E.A., Gherghiceanu F., Andronic O., Ion D., Stanescu M., Bolocan A. (2019). The use of nutritional supplement in romanian patients—Attitudes and beliefs. Farmacia.

[18] Hovanet M.V., Oprea E., Ancuceanu R., Duṭu L., Budura E.A., Seremet O., Ancu I., Moroṣan E. (2016). Wound Healing Properties of *Ziziphus jujuba* Mill. Leaves. Rom. Biotechnol. Lett..

[19] Gruszecka-Kosowska A. (2020). Human Health Risk Assessment and Potentially Harmful Element Contents in the Cereals Cultivated on Agricultural Soils. Int. J. Environ. Res. Public Health.

[20] National Academies of Sciences, Engineering, and Medicine (2016). Genetically Engineered Crops: Experiences and Prospects.

[21] Goesaert H., Brijs K., Veraverbeke W., Courtin C., Gebruers K., Delcour J. (2005). Wheat flour constituents: How they impact bread quality, and how to impact their functionality. Trends Food Sci. Technol..

[22] Cho I.H., Peterson D.G. (2010). Chemistry of bread aroma: A review. Food Sci. Biotechnol..

[23] Sabença C., Ribeiro M., Sousa T., Poeta P., Bagulho A.S., Igrejas G. (2021). Wheat/Gluten-Related Disorders and Gluten-Free Diet Misconceptions: A Review. Foods.

[24] Steiner E., Auer A., Becker T., Gastl M. (2012). Comparison of beer quality attributes between beers brewed with 100% barley malt and 100% barley raw material. J. Sci. Food Agric..

[25] Szambelan K., Nowak J., Szwengiel A., Jelen H. (2020). Comparison of sorghum and maize raw distillates: Factors affecting ethanol efficiency and volatile by-product profile. J. Cereal Sci..

[26] https://www.incda-fundulea.ro/fise/fise.html.

[27] Bradford M.M. (1976). A rapid and sensitive method for quantitation of microgram quantities of protein utilizing principle of protein- dye binding. Anal. Biochem..

[28] Kjeldahl J.Z. (1883). A new method for the determination of nitrogen in organic bodies. Anal. Chem..

[29] Council of Europe (2019). European Pharmacopoeia.

[30] Decker F. (2018). Gluten Content of Flours. https://healthyeating.sfgate.com/gluten-content-flours-11162.html.

[31] Schopf M., Wehrli M.C., Becker T., Jekle M., Scherf K.A. (2021). Fundamental characterization of wheat gluten. Eur. Food Res. Technol..

[32] Zilic S., Barac M., Pesic M., Dodig D., Ignjatovic-Micic D. (2011). Characterization of proteins from grain of different bread and durum wheat genotypes. Int. J. Mol. Sci..

[33] Branlard G., Dardevet M. (1985). Diversity of grain protein and bread wheat quality: II. Correlation between high molecular weight subunits of glutenin and flour quality characteristics. J. Cereal Sci..

[34] Magallanes-Lopez A.M., Ammar K., Morales-Dorantes A., Gonzalez-Santoyo H., Crossa J., Guzmán C. (2017). Grain quality traits of commercial durum wheat varieties and their relationships with drought stress and glutenins composition. J. Cereal Sci..

[35] Araya-Flores J., Guzman C., Matus I., Parada R., Jarpa G., de Camargo A.C., Shahidi F., Schwember A.R. (2020). New Findings in the Amino Acid Profile and Gene Expression in Contrasting Durum Wheat Gluten Strength Genotypes during Grain Filling. J. Agric. Food Chem..

[36] Siddiqi R.A., Singh T.P., Rani M., Sogi D.S., Bhat M.A. (2020). Diversity in Grain, Flour, Amino Acid Composition, Protein Profiling, and Proportion of Total Flour Proteins of Different Wheat Cultivars of North India. Front. Nutr..

[37] Xue C., Matros A., Mock H.P., Mühling K.H. (2019). Protein composition and baking quality of wheat flour as affected by split nitrogen application. Front. Plant Sci..

[38] Dhaka V., Khatkar B.S. (2015). Effects of gliadin/glutenin and HMW-GS/LMW-GS ratio on dough rheological properties and bread-making potential of wheat varieties. J. Food Qual..

[39] Tomić J., Torbica A., Popović L., Hristov N., Nikolovski B. (2016). Wheat breadmaking properties in dependance on wheat enzymes status and climate conditions. Food Chem..

[40] US Department of Agriculture (2022). Food Data Central Search Result for Wheat and Barley Flour.

[41] van Vliet S., Burd N.A., van Loon L.J. (2015). The skeletal muscle anabolic response to plant- versus animal-based protein consumption. J. Nutr..

[42] Gorissen S.H.M., Crombag J.J.R., Senden J.M.G., Waterval W.A.H., Bierau J., Verdijk L.B., van Loon L.J.C. (2018). Protein content and amino acid composition of commercially available plant-based protein isolates. Amino Acids.

[43] Ponzo V., Ferrocino I., Goitre I., Pellegrini M., Bruno M., Astegiano M., Cadario G., Castellana E., Bioletto F., Corvaglia M.R. (2021). Non-Celiac Gluten/Wheat Sensitivity: Clinical Characteristics and Microbiota and Mycobiota Composition by Response to the Gluten Challenge Test. Nutrients.

[44] Žilić S., Žilić S. (2013). Wheat Gluten: Composition and Health Effects. Gluten: Sources, Composition and Health Effects.

[45] Wang C., Tian Z., Zhao J., Chen L., Wang Y., Liu H. Electrophoretic Properties, Functionality of Barley Protein Isolates. Proceedings of the 2010 4th International Conference on Bioinformatics and Biomedical Engineering.

[46] Franco-Robles E., López M.G. (2015). Implication of fructans in health: Immunomodulatory and antioxidant mechanisms. Sci. World J..

[47] Fraberger L.-M.C.V. (2018). Applicability of Yeast Fermentation to Reduce Fructans and Other FODMAPs. Nutrients.

[48] Mititelu M., Stanciu T.I., Udeanu D.I., Popa D.E., Drăgănescu D., Cobelschi C., Grigore N.D., Pop A.L., Ghica M. (2021). The impact of Covid-19 lockdown on the lifestyle and dietary patterns among romanian population. Farmacia.

[49] Năstăsescu V., Mititelu M., Stanciu T.I., Drăgănescu D., Grigore N.D., Udeanu D.I., Stanciu G., Neacșu S.M., Dinu-Pîrvu C.E., Oprea E. (2022). Food Habits and Lifestyle of Romanians in the Context of the COVID-19 Pandemic. Nutrients.

[50] Hughes R.L., Alvarado D.A., Swanson K.S., Holscher H.D. (2021). The Prebiotic Potential of Inulin-type Fructans: A Systematic Review. Adv. Nutr. Int. Rev. J..

[51] Mietton L., Samson M.-F., Marlin T., Godet T., Nolleau V., Guezenec S., Segond D., Nidelet T., Desclaux D., Sicard D. (2022). Impact of Leavening Agent and Wheat Variety on Bread Organoleptic and Nutritional Quality. Microorganisms.

[52] Pico J., Bernal J., Gómez M. (2015). Wheat bread aroma compounds in crumb and crust: A review. Food Res. Int..

[53] Lau S.W., Chong A.Q., Chin N.L., Talib R.A., Basha R.K. (2021). Sourdough Microbiome Comparison and Benefits. Microorganisms.

[54] Dabija A., Ciocan M.E., Chetrariu A., Codină G.G. (2021). Maize and Sorghum as Raw Materials for Brewing, a Review. Appl. Sci..

[55] Chandra G.S., Proudlove M.O., Baxter E.D. (1999). The structure of barley endosperm an important determinant of malt modification. J. Sci. Food Agric..

[56] Konfo C.T.R., Chabi N.W., Dahouenon-Ahoussi E., Cakpo-Chichi M., Soumanou M.M., Sohounhloue D.C.K. (2020). Improvement of African traditional sorghum beers quality and potential applications of plants extracts for their stabilization: A review. J. Microbiol. Biotechnol. Food Sci..

[57] Mititelu M., Ghica M., Ionita A.C., Moroşan E. (2019). The influence of heavy metals contamination in soil on the composition of some wild edible mushrooms. Farmacia.

[58] Kopittke P.M., Menzies N.W., Wang P., McKenna B.A., Lombi E. (2019). Soil and the intensification of agriculture for global food security. Environ. Int..

[59] Ioniţă A.C., Ghica M., Moroşan E., Nicolescu F., Mititelu M. (2019). In vitro effects of some synthesized aminoacetanilide n’-substituted on human leukocytes separated from peripheral blood. Farmacia.

[60] Jassim Aziz R., Mihele D., Dogaru E. (2010). Study Regarding the Influence of *Vitis vinifera* Fruit (Muscat of Hamburg Species) on Some Biochemical Parameters. Farmacia.

